# Microstructure and Mechanical Properties of a Ti-Al-Mo-V-Cr-Sn-Zr Titanium Alloy via Double-Annealing Heat Treatment

**DOI:** 10.3390/ma19122553

**Published:** 2026-06-12

**Authors:** Jinfeng Shu, Bao Qu, Yingjie Ma, Kang Li, Fang Hao, Ning Zhao, Biao Ju, Yong Ren, Jing Yang, Tao Wang, Jinwen Lei, Xianghong Liu

**Affiliations:** 1 Northwest Institute for Non-Ferrous Metal Research, Xi’an 710016, China; 2 Western Superconducting Materials Technology Co., Ltd., Xi’an 710018, China; 3 Institute of Metal Research, Chinese Academy of Sciences, Shenyang 110016, China

**Keywords:** titanium alloy, double annealing, cooling rate, microstructure, mechanical properties

## Abstract

Achieving a favorable synergy of strength, ductility, and toughness is a critical challenge for expanding the engineering applications of titanium alloys. In this work, a medium-strength and high-toughness novel Ti-Al-Mo-V-Cr-Sn-Zr (named Ti62F) titanium alloy in the form of a Φ400 mm bar was adopted to systematically investigate the regulation behavior of double annealing on its microstructure and mechanical properties, and quantitative correlations between microstructural parameters and macroscopic properties were established. Increasing the cooling rate during the first annealing stage (air cooling, force air cooling and water quenching) significantly refined the secondary α (α_s_) phase and reduced the volume fraction and size of the primary α (α_p_) phase, leading to an increase in the ultimate tensile strength of the alloy from 1077 MPa to 1229 MPa. However, the impact-absorbed energy decreased from 51.5 J to 23.3 J. When the second annealing temperature was varied within the range of 625–675 °C, the ultimate tensile strength fluctuated slightly and the impact toughness increased moderately. Equiaxed α_p_ phase and relatively thick α_s_ can induce multiple crack deflections, prolong the crack propagation path and enhance energy absorption. Dislocations are mainly piled up at α/β phase boundaries, triggering void nucleation and growth, which dominate the ductility and toughness levels. Tensile twinning acts only as an auxiliary deformation mechanism and contributes limitedly to toughness. After heat treatment under the optimized schedule of 880 °C/2 h/AC + 650 °C/4 h/AC, the Ti62F alloy exhibits a superior strength–toughness balance compared with conventional medium-strength titanium alloys such as TA15, TC4, and TC4-DT. The findings can provide a heat treatment basis for microstructural regulation of large-size Ti62F bars and their engineering applications in aerospace structural components.

## 1. Introduction

Titanium alloys have attracted considerable attention due to their low density, high strength at room and elevated temperatures, and excellent oxidation resistance, meeting the rapidly growing demand for lightweight design in the aerospace industry [1,2,3]. Compared with high-strength titanium alloys, medium-strength titanium alloys with a strength range of 800–1000 MPa are widely used in aerospace structural components owing to their superior ductility and toughness, such as TA15 (Ti-6.5Al-2Zr-1Mo-1V), TC4 (Ti-6Al-4V), and TC4-DT titanium alloys [4,5,6]. Therefore, the development of medium-strength titanium alloys and the achievement of a favorable strength–ductility–toughness synergy are beneficial for further expanding their engineering applications. Ti62F titanium alloy is a novel medium-strength, high-toughness alloy developed by the Institute of Metal Research, Chinese Academy of Sciences. Aluminum acts as an α-stabilizing element to enhance strength and thermal stability, and its content is generally controlled below 6% to avoid the formation of a brittle Ti_3_Al phase that would deteriorate ductility [7,8]. Mo and V serve as eutectoid β-stabilizing elements to improve strength and corrosion resistance, while Cr is a weakly eutectoid β-stabilizing element used to balance strength and ductility while maintaining excellent toughness [9,10]. Sn and Zr are neutral elements typically added to provide supplementary strengthening [11]. The yield strength of Ti62F alloy ranges from 900 to 1000 MPa, with a service temperature less than 450 °C. It can be applied to structural components such as fuselage frames and beams in advanced aircraft, effectively improving the safety and service life of key components.

The development of impact toughness research largely originated from investigations into catastrophic structural failures, such as the sinking of the *Titanic* (1912), the second collapse of the *Quebec Bridge* (1916), and fractures of *Liberty* ships during World War II (1940s). Consequently, impact toughness has become an essential mechanical property, comparable to strength and ductility, in various structural applications. However, the impact toughness of common commercial titanium alloys is lower than that of steels with equivalent strength levels [12]. Complicating matters further, the conventional strengthening mechanisms in metals rely on impeding dislocation motion, which typically leads to a significant loss in toughness. For some titanium alloys, a 10% increase in strength is accompanied by a reduction in impact toughness exceeding 30%. For instance, when the strength of TC4 titanium alloy was increased from 881 MPa to 975 MPa via heat treatment adjustment, its impact toughness decreased from 85 J·cm^−2^ to 50 J·cm^−2^ [13]. Similarly, as the strength of TC21G titanium alloy rose from 1048 MPa to 1331 MPa, impact toughness dropped from 53 J·cm^−2^ to 29 J·cm^−2^ [14]. This strong strength–toughness trade-off is even more pronounced in high-strength titanium alloys [15,16]. Therefore, achieving a favorable strength–ductility–toughness balance is critical for the widespread engineering application of titanium alloys.

Heat treatment processes significantly influence the microstructure of titanium alloys, and microstructural evolution is the key to achieving a favorable strength–ductility–toughness balance. Grain refinement has been adopted as an effective strategy to improve the comprehensive strength–ductility–toughness of titanium alloys, owing to the fact that high-density grain boundaries can impede dislocation motion while alleviating stress concentration [17,18]. However, this approach is difficult to implement in the production of large-scale bars due to their dimensions and repeated heating. Numerous experimental results indicate that fully lamellar or basket-weave microstructures can promote crack deflection and thus enhance impact toughness, but at the cost of a considerable reduction in ductility [19,20]. Bimodal microstructures consisting of equiaxed αp and transformed β (β_t_) have attracted extensive attention. Such microstructures improve overall deformation uniformity and increase resistance to crack initiation and propagation by facilitating the formation of a wider plastic zone at the crack tip [21,22,23]. In addition, bimodal microstructures combine the ductility of equiaxed microstructures and the strength of lamellar microstructures, making them the preferred microstructure for achieving balanced strength–ductility–toughness in titanium alloys.

For large-scale titanium alloy bars, the morphological characteristics of bimodal microstructures are strongly dependent on cooling rate. As a newly developed medium-strength and high-toughness titanium alloy, research on the influence of heat treatment on the microstructure and mechanical properties of the Ti62F alloy remains very limited. This work systematically investigates the effects of the cooling rate after the first annealing and the temperature of the second annealing on the microstructure and mechanical properties of the Ti62F alloy under various dual-annealing conditions. Based on experimental results, quantitative relationships between microstructural parameters and macroscopic mechanical properties were established. The study on achieving a favorable strength–ductility–toughness balance in this alloy possesses significant engineering value for its application in structural components in the aerospace field.

## 2. Materials and Methods

### 2.1. Materials

**Figure 1:**
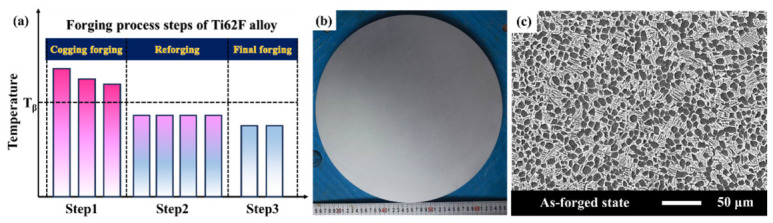
Free forging process steps and microstructure of forged material. (**a**) Free forging process steps; (**b**) macrostructure; (**c**) microstructure.

The chemical composition of the Ti62F titanium alloy is shown in Table 1. The as-received material used in this paper was provided by Western Superconducting Materials Technology Co., Ltd. (Xi’an, China). The raw material was a large-scale bar with a diameter of Φ400 mm, fabricated via vacuum arc melting followed by free forging. The free forging procedure, as illustrated in Figure 1a, consisted of three stages: cogging forging, reforging in the two-phase region, and finial forging. The phase transformation temperature of the alloy was determined to be 920 °C by metallographic examination. The macrostructure and microstructure of the as-forged bar cross-section are presented in Figure 1b. The uniform and indistinct grain pattern in the macrostructure confirmed the homogeneous microstructure of the bar. As shown in Figure 1c, the microstructure exhibited a bimodal microstructure composed of equiaxed α_p_ phase and lamellar α_s_ phase.

### 2.2. Double-Annealing Heat Treatment Process

**Figure 2:**
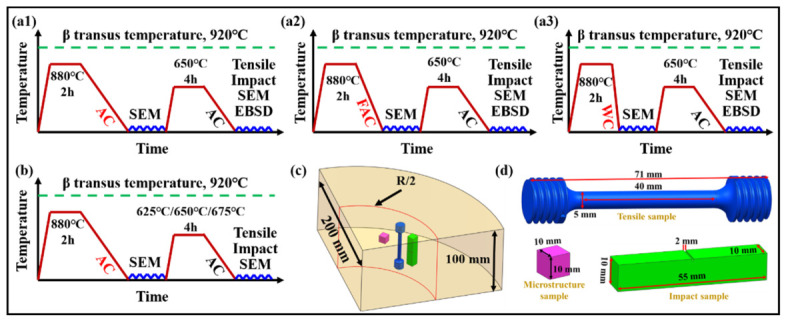
Heat treatment routines, diagram of sampling locations and sample size. (**a1**–**a3**,**b**) Heat treatment routines; (**c**) diagram of sampling locations; (**d**) sample size.

Cylindrical specimens with a length of 100 mm were sectioned from the as-forged bar and divided into four equal parts, as shown in Figure 2c, for subsequent dual-annealing experiments. For bulk heat treatment, it is essential to ensure the material is fully heated through. The duration is typically determined by empirical formula derived from titanium alloy forging practices. The heat treatment schedules designed in this work are illustrated in Figure 2(a1–a3),b. For investigating the effect of cooling rate on mechanical properties, the first annealing was conducted at 880 °C for 2 h, followed by three distinct cooling rates: air cooling (AC), forced air cooling (FAC), and water cooling (WC), respectively. Subsequently, the second annealing was performed at 650 °C for 4 h, followed by air cooling to room temperature. The heat treatment route shown in Figure 2b was employed to study the influence of the second annealing temperature, in which the second annealing was carried out at 625 °C, 650 °C, and 675 °C for 4 h, respectively, with air cooling as the uniform cooling method.

### 2.3. Properties and Microstructure Characterization

Due to the difference in cooling rates between the core and the surface of the block materials, the strength is generally slightly lower than that of surface. Therefore, the performance of the core is analyzed in this paper, that is, at R/2 position in Figure 2c. To ensure the comparability of the experiment, the sampling positions were kept consistent across all heat treatment conditions. Room-temperature tensile tests were carried out on an Instron 5982 universal testing machine at a crosshead speed of 1 mm/min. The cylindrical tensile specimens had a total length of 71 mm, a gauge length of 40 mm, and a diameter of 5 mm. Charpy impact tests were performed on the Charpy impact tester using U-notched specimens with a notch radius of 1 mm. The specimens were 10 mm in width, 10 mm in thickness, and 55 mm in length. Schematic diagrams of the sampling positions and detailed dimensions are shown in Figure 2c,d. The core area usually has the lowest strength after heat treatment. Therefore, by evaluating the material performance based on the core properties, it can effectively reflect the key service conditions of the block materials. To ensure the reliability of experimental data, at least three replicate tests were conducted for each heat treatment condition. Three parallel specimens were tested for each group, and the arithmetic mean was adopted as the representative value. Error bars in all figures indicate data variation: the upper deviation is defined as the difference between the maximum value and the mean, while the lower deviation refers to the difference between the mean and the minimum value.

Microstructural characterization was performed using optical microscopy (OM), a JEOL IT700 scanning electron microscope (SEM) (Tokyo, Japan) and electron backscatter diffraction (EBSD). Specimens for OM and SEM observation were prepared by grinding with silicon carbide abrasive papers and mechanical polishing, followed by etching with Kroll’s reagent. The EBSD specimens were prepared by electrolytic polishing.

## 3. Results

### 3.1. Microstructure After Different Heat Treatment Processes

**Figure 3:**
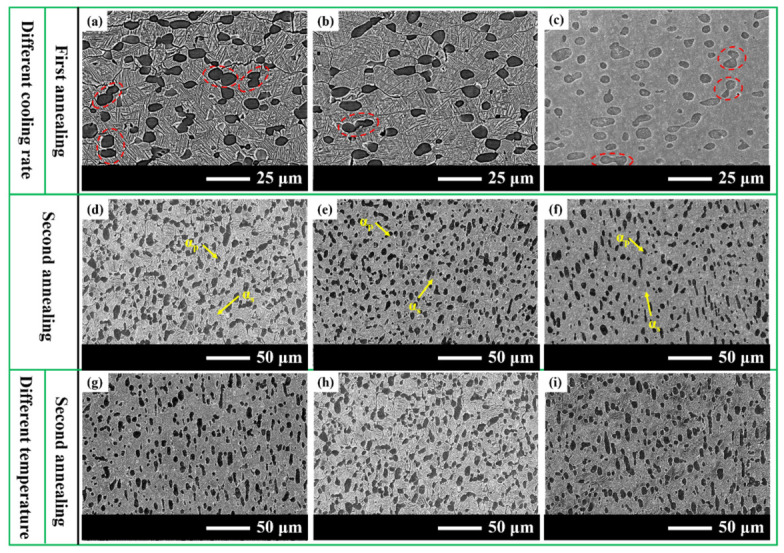
Microstructure after different heat treatment conditions. (**a**–**c**) 880 °C/2 h followed by AC, FAC and WC; (**d**,**h**) 880 °C/AC + 650 °C/AC; (**e**) 880 °C/FAC + 650 °C/AC; (**f**) 880 °C/WC + 650 °C/AC; (**g**) 880 °C/AC + 625 °C/AC; (**i**) 880 °C/AC + 675 °C/AC.

The microstructure after the first annealing with different cooling rates is shown in Figure 3a–c. The thickness of lamellar and the size and volume of α_p_ decrease with the increase in cooling rate. The lamellar structure formed after water cooling was too fine to be clearly resolved by SEM. It is noted that some “peanut” shape of the α phase formed, which is identified by red circles in the figure. This is due to the thermal groove at the phase boundaries that can be interpreted as the mechanism of boundary splitting [24,25]. The main process involves the formation of extensive dislocation tangles and subgrain boundaries within the lamellar microstructure. Thermal grooves then develop at the newly formed α/β phase boundaries, and the β phase diffuses along the subgrain boundaries into the lamellar α phase. During the diffusion process, the smaller lamellar α gradually dissolve while the larger lamellar α gradually coarsen. Subsequently, the coarsening α is globularized by boundary splitting [26,27]. Therefore, the boundary splitting plays an important role in the globularization of the lamellar α phase. The microstructure of the sample after the second annealing in the heat treatment condition of 650 °C/4 h/AC is shown in Figure 3d–f. The thickness of the lamellar α phase is coarsening, especially the sample with water cooling according to Figure 3c,f. Furthermore, the thickness of the lamellar α phase first increases and then tends to be constant with the increase in the second annealing temperature as shown in Figure 3g–i.

### 3.2. Tensile and Impact Properties After Different Heat Treatment Processes

**Figure 4:**
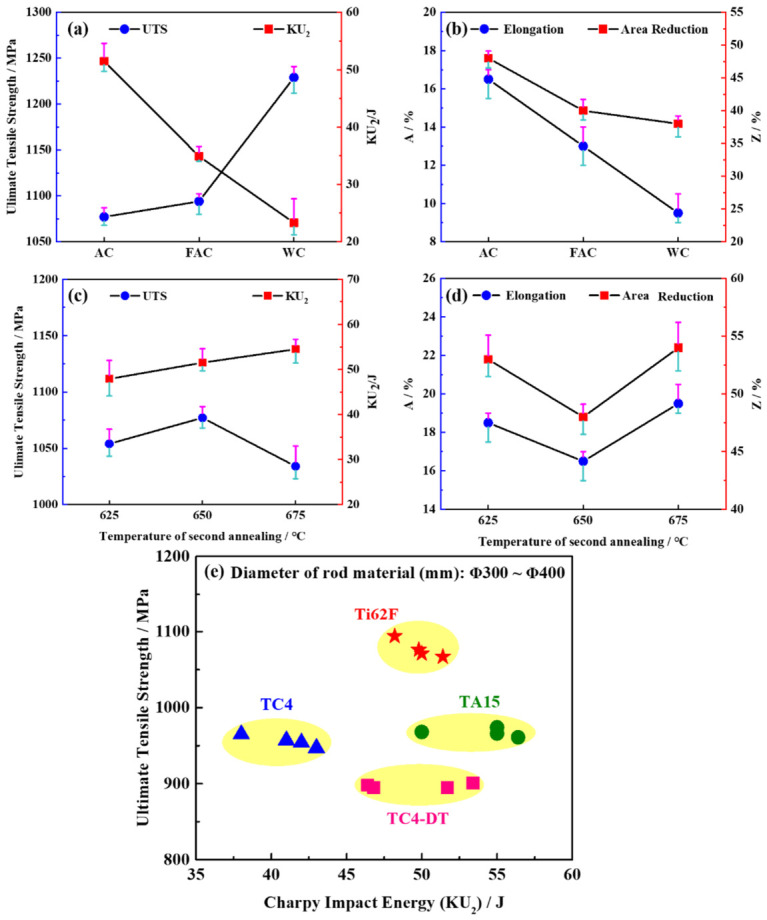
Tensile and impact properties of different heat treatment conditions and comparison of typical medium-strength titanium alloy properties. (**a**,**b**) Ultimate tensile strength, impact-absorbed energy, elongation and area reduction in the Ti62F alloy under different cooling modes (AC, FAC, WC) after first annealing at 880 °C/2 h, followed by 650 °C/4 h/AC. (**c**,**d**) Properties at different secondary annealing temperatures (625 °C, 650 °C, 675 °C) after 880 °C/2 h/AC. (**e**) Strength–toughness comparison between the developed Ti62F alloy (red star) and conventional TC4 (blue triangle), TA15 (green circle), and TC4-DT (pink square) alloys.

The tensile and impact properties of different cooling rates are shown in Figure 4a,b. The ultimate tensile strength increases from 1077 MPa (AC) to 1229 MPa (WC) with the increase in cooling rate, while the impact energy shows a linear descent from 51.5 J to 23.3 J. The total elongation and area reduction gradually decrease with the increase in cooling rate. The main reason for the increase in strength is the decrease in the thickness of lamellar α as shown in Figure 3d–f. Based on the influence of cooling rate on performance, the AC method was selected to conduct the research on the second annealing temperature. In Figure 4c,d, with the increase in the second annealing temperature, the ultimate tensile strength first increased and then decreased, while the impact energy shows a continuous slightly upward trend from 47.9 J to 54.5 J due to the increase in the thickness of the lamellar α [28]. The total elongation and area reduction are in an inverse relationship with the tensile strength by analyzing Figure 4c,d. Compared with other typical medium-strength titanium alloys, such as TA15, TC4 and TC4-DT titanium alloys, the Ti62F titanium alloy has higher ultimate tensile strength and impact toughness as shown in Figure 4e, which demonstrates the Ti62F titanium alloy has a much broader application potential.

### 3.3. Fracture Morphology

#### 3.3.1. Fracture Morphology of Tensile

**Figure 5:**
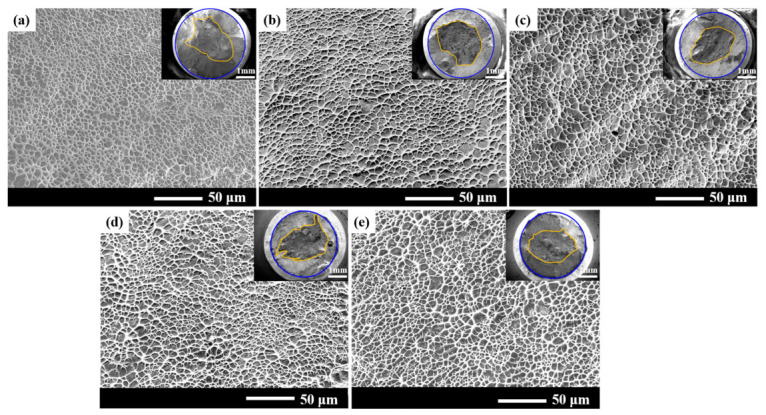
Fracture morphology after tensile test with different heat treatment conditions. (**a**) 880 °C/2 h/WC + 650 °C/4 h/AC; (**b**) 880 °C/2 h/FAC + 650 °C/4 h/AC; (**c**) 880 °C/2 h/AC + 650 °C/4 h/AC; (**d**) 880 °C/2 h/AC + 625 °C/4 h/AC; (**e**) 880 °C/2 h/AC + 675 °C/4 h/AC.

The depth and size of dimples on the tensile fracture surfaces gradually decrease with increasing cooling rate, as shown in Figure 5a–c. In addition, samples with higher ductility exhibit obvious necking and a larger proportion of shear lips, as can be seen by comparing Figure 5a,c. In general, the β phase with a bcc crystal structure can accommodate greater plastic deformation compared with the α phase with an hcp crystal structure. The difference in resistance to plastic deformation between the α and β phases leads to stress concentration at the α/β interfaces, inducing microvoid nucleation at these boundaries. These microvoids then coalesce and gradually grow into macroscopic cracks [29]. For samples heat-treated at 625 °C and 675 °C, the dimple size and depth are less uniform than those at 650 °C, but the overall average dimple dimensions are similar, and the proportions of shear lips are also comparable. Therefore, the effect of the second annealing temperature on elongation is less significant than that of the cooling rate of first annealing. The total elongations are 17.5%, 16.5%, and 18.0%, respectively.

#### 3.3.2. Fracture Morphology of Impact

**Figure 6:**
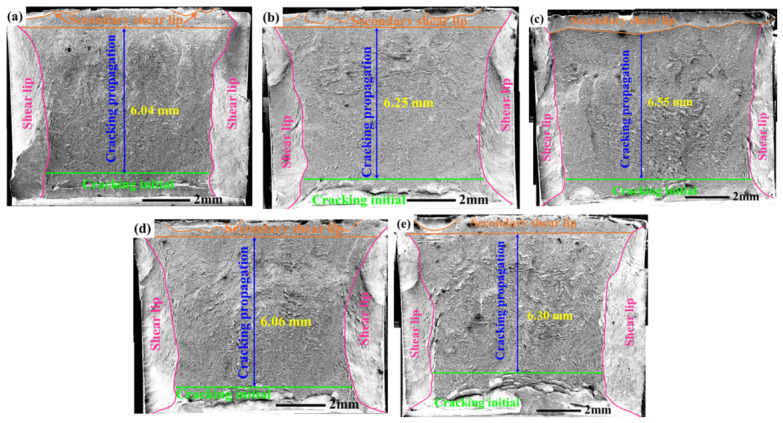
Fracture morphology after impact toughness with different heat treatment conditions. (**a**) 880 °C/2 h/AC + 650 °C/4 h/AC; (**b**) 880 °C/2 h/FAC + 650 °C/4 h/AC; (**c**) 880 °C/2 h/WC + 650 °C/4 h/AC; (**d**) 880 °C/2 h/AC + 625 °C/4 h/AC; (**e**) 880 °C/2 h/AC + 675 °C/4 h/AC.

The impact fracture surface generally consists of a crack initiation zone, a crack propagation zone, and a shear lip zone, and occasionally complete or discontinuous secondary shear lips may appear [30]. With increasing cooling rate, the crack propagation zone gradually expands while the shear lip zone shrinks, and the number and depth of cracks in the crack initiation zone gradually decrease, as illustrated in Figure 6a–c. A larger shear lip zone indicates more pronounced plastic deformation, while more and deeper cracks in the crack initiation zone imply greater energy absorption during crack nucleation. A larger crack propagation zone suggests that the crack propagates rapidly during impact, absorbing less energy. Although the WC specimen in Figure 6c exhibits a complete area of secondary shear lips, this cannot compensate for the toughness reduction caused by the reduced shear lip area and enlarged crack propagation zone. With increasing second annealing temperature, the shear lip regions of the fracture surfaces are comparable, while the differences in the crack initiation zones are significant, as shown in Figure 6a,d,e. Distinct cracks in the crack initiation zone, as observed in Figure 6e, indicate higher energy consumption during crack nucleation. Therefore, the decrease in impact toughness with increasing cooling rate is mainly attributed to the reduction in the proportion of shear lips, whereas the slight increase in impact toughness with rising second annealing temperature is primarily caused by the greater number and depth of cracks in the crack initiation zone.

## 4. Discussion

### 4.1. Effect of Heat Treatment Process on Microstructure

**Figure 7:**
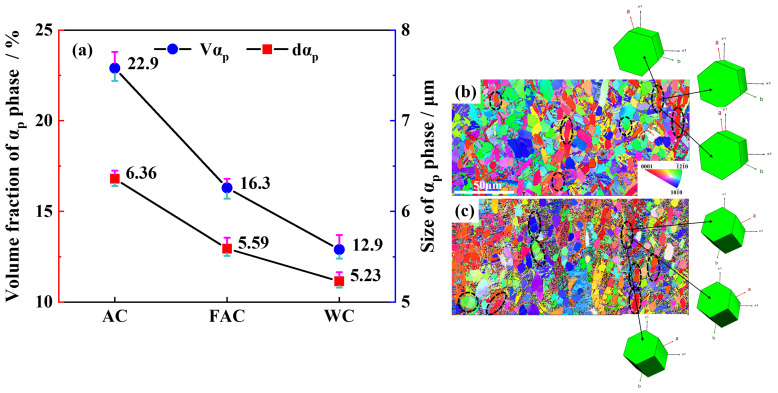
Volume fraction and size of α_p_ phase and IPF with different cooling rates. (**a**) The volume fraction and size of α_p_ phase; (**b**) 880 °C/2 h/AC + 650 °C/4 h/AC; (**c**) 880 °C/2 h/WC + 650 °C/4 h/AC.

With increasing cooling rate, the volume fraction and average size of the α_p_ phase decrease approximately linearly, as shown in Figure 7a. The volume fractions of α_p_ under AC, FAC, and WC are 22.9%, 16.3%, and 12.9%, respectively, while the average sizes of αp are 6.36 μm, 5.59 μm, and 5.23 μm, respectively. The main reason is that, compared with the WC sample, the AC sample allows a longer growth time for the α_p_ phase, and promotes more uniform and sufficient elemental diffusion. Figure 7b,c show the inverse pole figures (IPFs) of specimens after first annealing via AC and WC, respectively, followed by second annealing at 650 °C for 4 h with AC. As indicated by the black dashed lines in the figures, subgrain boundaries form within some α_p_ phase, and their similar crystallographic orientations suggest that dislocation recovery occurs in the α_p_ phase during heat treatment, gradually leading to the formation of subgrain boundaries [5].

**Figure 8:**
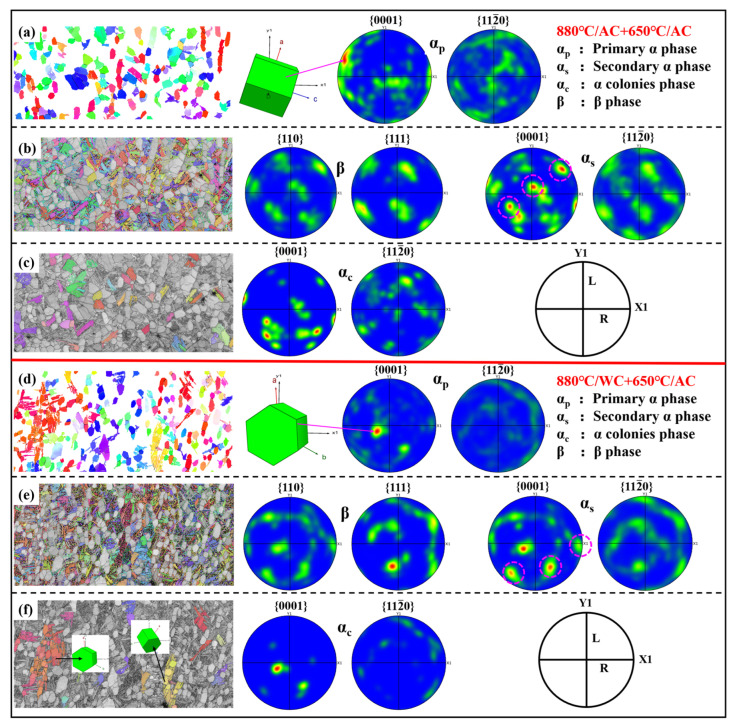
IPF and IF of αp phase, αs phase and αc of different cooling rates. (**a**–**c**) 880 °C/2 h/AC + 650 °C/4 h/AC; (**d**–**f**) 880 °C/2 h/WC + 650 °C/4 h/AC.

Figure 8 shows the IPFs and pole figures (PFs) of specimens after first annealing via AC and WC, including α_p_, α_s_, and α_c_. In both specimens, the *c* axis of the α phase with *hcp* crystal structure tends to be parallel to the radial direction of the bar, exhibiting a pronounced axial texture, as shown in Figure 8a,d. The precipitation of the α_s_ phase follows the Burgers orientation relationship with the parent β grain, as illustrated in Figure 8b,e: (110)_β_//(0001)_α_, [111]_β_//[$11\overset{-}{2}0$]_α_. Notably, the two specimens differ significantly in the orientation distribution of α_c_. In the AC sample, α_c_ does not show a strong orientation similarity to α_p_ and is relatively dispersed. This promotes a more tortuous crack propagation path and enhances energy absorption [31]. In contrast, in the WC sample, the orientations of α_c_ and α_p_ are nearly consistent, and α_s_ also exhibits orientations close to those of α_p_, as shown in Figure 8d,f. Cracks can propagate directly through α_c_ and α_p_ phases, resulting in a straight crack propagation path and consequently reduced impact toughness [32].

**Figure 9:**
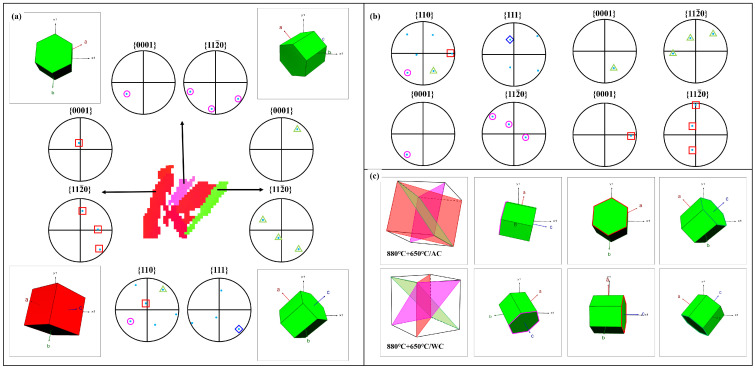
Pole figure of each variant and 3D schematic illustration of orientation relationships among the variants and β grain of different cooling rates. (**a**) Pole figure of 880 °C/2 h/AC + 650 °C/4 h/AC; (**b**) pole figure of 880 °C/2 h/WC + 650 °C/4 h/AC; (**c**) 3D schematic illustration of orientation relationships among the variants and β grain.

Further analysis was performed on the αs phase in the AC and WC specimens, focusing on the orientations enclosed by the pink dashed circles in the IPFs of Figure 8b,e. The IPFs of three typical α_s_ orientations for both AC and WC specimens are presented in Figure 9. As observed in Figure 9a,b, the variants form a large Type I cluster that shares a common <$11\overset{-}{2}0$> α pole and exhibits a 60° misorientation in the {0001} inverse pole figure. This clustering arises from the self-accommodation mechanism of strain energy during phase transformation [33]. Related studies have shown that Type I and Type II clusters minimize shear element of the transformation strain, and Type I clusters are identified as the preferred cluster configuration when both dilatational and shear elements are considered [34,35]. In addition, for the AC specimen, such variant selection is not only related to self-accommodation but also associated with the equiaxed α_p_ phase. As shown in Figure 8d,f, the equiaxed α_p_ phase exhibits orientations closely similar to those of the α_s_ phase. According to relevant report, this phenomenon arises from the short-range diffusion of atoms at interfaces that facilitates the β → α phase transformation with a higher transformation rate. The α phase nucleates and grows at the boundaries, forming α_c_ with numerous adjacent α variants of similar orientation, leading to strong variant selection [36].

### 4.2. Effect of Heat Treatment Process on Tensile Properties

**Figure 10:**
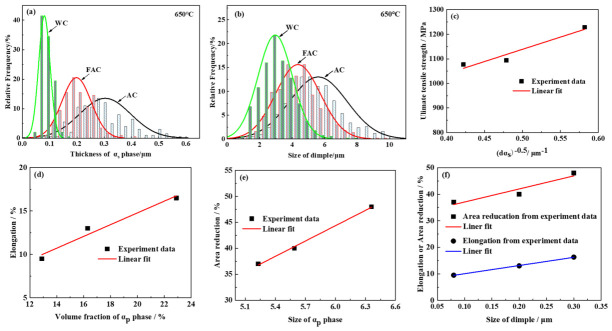
Thickness of lamellar α_s_, size of dimple and correlation between microstructure variables and strength-plasticity. (**a**) Thickness of lamellar α_s_; (**b**) size of dimple; (**c**) strength and lamellar thickness; (**d**) elongation and volume fraction of α_p_ phase; (**e**) area reduction and size of α_p_ phase; (**f**) elongation, area reduction and size of dimple.

With increasing cooling rate, both the lamellar thickness of the αs phase and the dimple size in the shear lip zone gradually decrease. Normal distribution plots were obtained based on analysis of 500 measurements as shown in Figure 10a,b. The average lamellar thicknesses under AC, FAC, and WC are 5.61 μm, 4.36 μm, and 2.95 μm, respectively, while the average dimple sizes are 0.30 μm, 0.20 μm, and 0.08 μm, correspondingly. Microstructural parameters exhibit an approximately linear relationship with strength and ductility, as illustrated in Figure 10c–e. Similarly, the dimple size in the shear lip zone shows an approximately linear correlation with ductility, as presented in Figure 10f. In Figure 10c, the relationship between strength and lamellar thickness follows the Hall–Petch relation [37]. Increasing the volume fraction and grain size of the equiaxed αp phase contributes to improved elongation and area reduction. The cooling method after the first annealing plays a critical role, as it exerts a strong influence on microstructural characteristics as shown in Figure 3. According to Figure 4c,d, the effect of the second annealing temperature is relatively mild. Therefore, adopting AC or FAC after the first annealing enables a favorable strength–ductility balance in the Ti62F alloy. For large-scale bars (Φ400 mm in this work), FAC is technically challenging, even more complex than WC. Considering engineering applications, AC is simpler and more feasible.

### 4.3. Effect of Heat Treatment Process on Impact Properties

**Figure 11:**
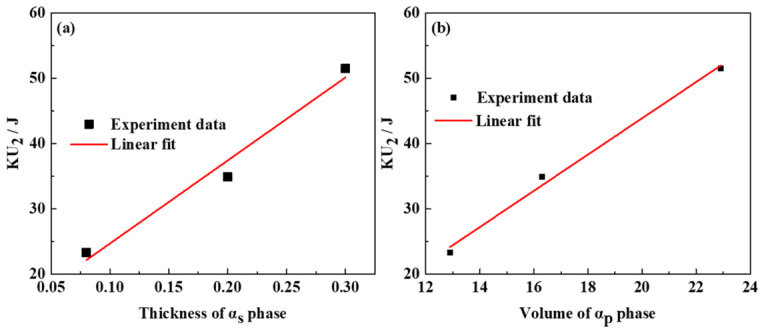
Correlation between thickness of α_s_, volume fraction of α_p_ and impact-absorbed energy. (**a**) Thickness of lamellar α_s_ and KU_2_; (**b**) volume fraction of α_p_ phase and KU_2_.

**Figure 12:**
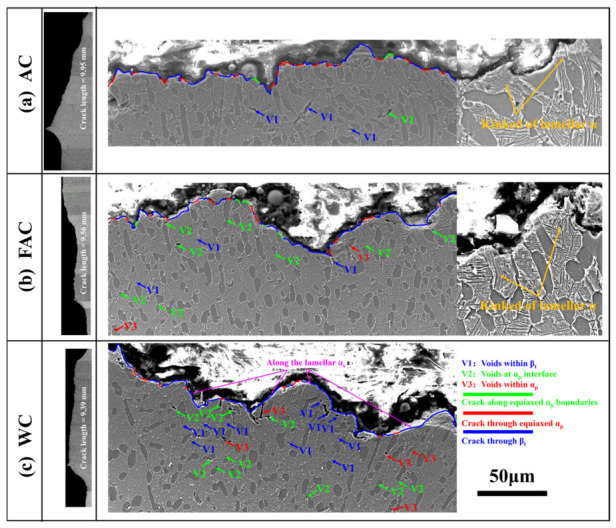
Crack length and crack propagation of the samples of different cooling rates. (**a**) 880 °C/2 h/AC + 650 °C/4 h/AC; (**b**) 880 °C/2 h/FAC + 650 °C/4 h/AC; (**c**) 880 °C/2 h/WC + 650 °C/4 h/AC.

The relationships between the thickness of the α_s_ phase, the volume fraction of the α_p_ phase, and the impact-absorbed energy are presented in Figure 11a,b. The impact-absorbed energy increases correspondingly with the increase in the lamellar thickness of the αs phase and the volume fraction of the αp phase. When the lamellar thickness in the transformed β (βt) region is fine, and the volume fraction and size of the α_p_ phase are small, cracks tend to propagate easily along the boundaries of the lamellar α_s_ phase and directly through the equiaxed α_p_ phase, as illustrated in Figure 12c. This results in a straighter crack propagation zone on the impact fracture surface, which exerts a detrimental effect on impact toughness [16]. Under AC, a large number of lamellar α_s_ phases and equiaxed α_p_ phases are interpenetrated and interconnected, as shown in Figure 12a. This microstructure induces multiple crack deflections, leading to a more tortuous crack propagation path and consequently improved impact toughness. In addition, in WC specimens, α colonies, primary and secondary α, develop a strong axial texture with uniform orientations in Figure 8. Cracks propagate linearly along oriented grains and worsen toughness. In AC samples, dispersed orientations cause frequent crack deflection and better toughness. Overall, microstructural refinement mainly accounts for the toughness reduction, and texture evolution aggravates this trend.

The impact-absorbed energies under AC, FAC, and WC are 51.5 J, 34.9 J, and 23.3 J, respectively. The microstructures of the crack propagation zones in the cross-section of impact specimens were characterized, as shown in Figure 12a–c. The green solid lines denote crack propagation along the boundaries of equiaxed α_p_, the red solid lines represent cracks penetrating through the equiaxed α_p_ phase, and the blue solid lines indicate cracks passing through the β_t_ region. V1 refers to voids formed inside of β_t_, V2 represents voids at the α_p_/β_t_ interface, and V3 denotes voids within the α_p_ phase. It can be seen that under all three cooling conditions, crack propagation mainly occurs by penetration through equiaxed α_p_ and β_t_. Crack propagation along grain boundaries occurs only in small equiaxed α_p_ grains and accounts for a small proportion, as shown in Figure 12a,b. With increasing cooling rate, the proportion of cracks penetrating βt increases, which is related to the highest volume fraction of β_t_ under water cooling. In addition, distorted coarse lamellar structures were observed in AC and FAC specimens to accommodate plastic deformation, whereas in WC specimens, cracks directly pass through the fine lamellar structures due to their small size.

Crack deflection is quantified by the ratio of actual crack length to theoretical straight crack length. The theoretical linear crack length of impact specimens is 8 mm. With actual crack lengths of 9.95 mm, 9.56 mm and 9.39 mm, the corresponding length ratios are 1.244, 1.195 and 1.174, respectively. A larger ratio represents more tortuous crack propagation and stronger crack deflection, which corresponds to higher impact-absorbed energy. The number and nucleation sites of voids significantly affect the crack propagation path and thus further influence the impact toughness of the alloy. Generally, a smaller number of voids corresponds to higher impact toughness [38], which is consistent with the observation that a faster cooling rate leads to lower impact toughness and a greater number of voids, as shown in Figure 12a–c. In the AC specimen, voids are mainly concentrated inside the β_t_ region, with only a small number appearing at α_p_ interfaces. In the FAC and WC specimens, voids form both inside β_t_ and at β_t_/α_p_ interfaces, whereas voids within the αp phase are much fewer. The lamellar structure and α colonies maintain the Burgers orientation relationship with the parent β grain, as illustrated in Figure 8 and Figure 9. Previous studies have reported that dislocations can transfer across adjacent α lamellae and colonies along several specific slip systems [39]. Notably, α colonies within the parent β grains also introduce additional α/β interfaces, promoting dislocation pile-up at these boundaries and consequently leading to the formation of V1-type voids [31]. The equiaxed αp phase is surrounded by hard β_t_ or α_p_ grains with different orientations, forming high-angle grain boundaries that hinder dislocation transmission across interfaces and cause dislocation pile-up. Subsequent stress concentration induces the formation of V2 and V3 voids at β_t_/α_p_ and α_p_/α_p_ interfaces, respectively [17].

**Figure 13:**
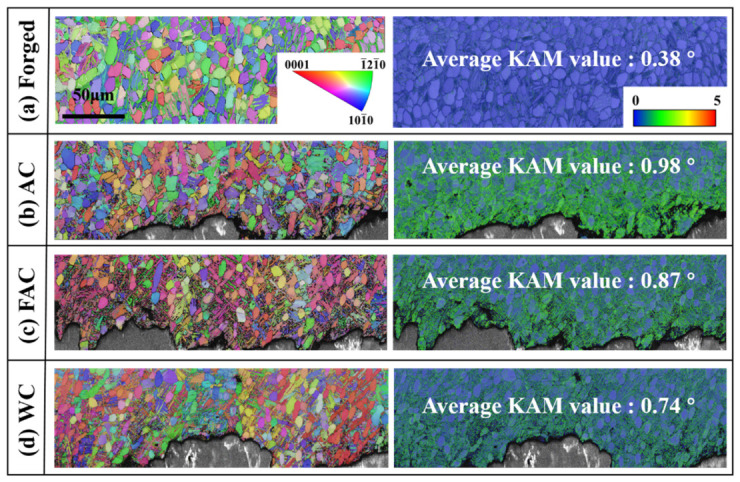
Inverse pole figure and KAM map of the samples of as-forged and different cooling rates. (**a**) As-forged state; (**b**) 880 °C/2 h/AC + 650 °C/4 h/AC; (**c**) 880 °C/2 h/FAC + 650 °C/4 h/AC; (**d**) 880 °C/2 h/WC + 650 °C/4 h/AC.

The characterization results of the regions near fracture in AC, FAC, and WC specimens are presented in Figure 13. The KAM maps show that the α_p_ phase exhibits low KAM values, while high KAM values are mainly concentrated at α_p_ boundaries and within the βt region. This indicates that dislocation pile-up tends to occur preferentially at interfaces, inducing local stress concentration, which is consistent with the high proportion of V1 and V2 voids observed in Figure 12. The as-forged state shows the lowest average KAM value of 0.38°. All three specimens exhibit relatively uniform KAM distributions in the regions beneath the fracture surface, but distinct differences in magnitude are evident. With decreasing cooling rate, the average KAM value increases gradually, reaching 0.74°, 0.87°, and 0.98° for WC, FAC, and AC specimens, respectively. Since KAM reflects local plastic strain and dislocation density, higher KAM values indicate better deformation coordination and higher plastic indicators, corresponding to the gradual enhancement of impact toughness [31].

**Figure 14:**
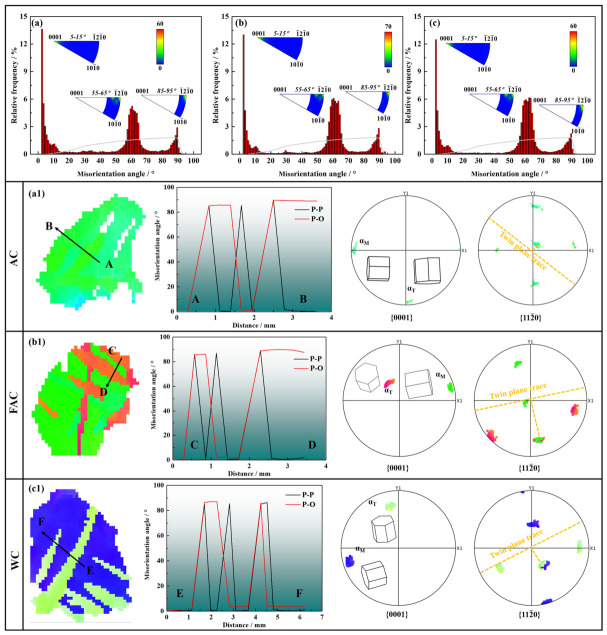
Misorientation angles, deformation twins, misorientation profiles, orientation of matrix and twinning in {0001} and {$10\overset{-}{1}$2}} pole figures of different cooling rates. (**a**,**a1**) 880 °C/2 h/AC + 650 °C/4 h/AC; (**b**,**b1**) 880 °C/2 h/FAC + 650 °C/4 h/AC; (**c**,**c1**) 880 °C/2 h/WC + 650 °C/4 h/AC.

Figure 14a–c display the orientation misorientation angle distributions and IPF of the specimens under three cooling rates. After impact testing, the fraction of low-angle grain boundaries increases slightly with decreasing cooling rate, indicating a gradual increase in plastic deformation that induces grain boundary rotation. Notably, a prominent peak appears at 60° ± 5° in all three specimens, which is attributed to the misorientation angles between different α variants within the same or similarly oriented parent β grain being close to (60°/[$11\overset{-}{2}0$], 60.83°/[$\overset{-}{1.377}$ $\overset{-}{1}$ 2.377 0.359], 63.26°/[$\overset{-}{10}\;5\;5\;\overset{-}{3}$]) [40,41]. A high pole density point appears at 85° ± 5° around the <$1\;1\;\overset{-}{2\;}0$> axis in the IPF, which arises from the activation of deformation twinning [42,43]. As shown in Figure 14a1–c1, the IF reveals that the α matrix (α_M_) and twinned α (α_T_) share a common plane. The trace of the twin plane is marked by orange dashed lines, indicating that the twin corresponds to the {$10\overset{-}{1}$2} <$11\overset{-}{2}0$> tensile twin type. During impact deformation of titanium alloys, twins accommodate c-axis strain, relieve stress concentration and blunt crack tips, thereby effectively improving impact toughness. Dense twin boundaries refine the microstructure, increase crack propagation resistance and absorb more impact energy [17]. Since the same twin type is activated with similar area fractions—4.9%, 5.7%, and 5.9% for AC, FAC, and WC specimens, respectively—twinning is regarded as an auxiliary deformation mechanism.

## 5. Conclusions

In this work, a novel medium-strength and high-toughness Ti62F titanium alloy in large-scale bar form was employed to systematically investigate the regulation behavior of double annealing on its microstructure and mechanical properties. The main conclusions are as follows:

1. Double annealing can effectively tailor the bimodal microstructure of the Φ400 mm large-size Ti62F titanium alloy, in which the cooling rate during the first annealing plays a dominant role. With increasing cooling rate (air cooling, force air cooling, water cooling), the volume fraction and size of primary α phase are significantly reduced, and the secondary α lamellae are remarkably refined. The second annealing temperature (625~675 °C) mainly affects the coarsening behavior of secondary α lamellae, and its effect on microstructural morphology is weaker than that of the cooling rate.
2. Room-temperature tensile properties exhibit a quantitative linear correlation with the microstructure. Increasing the cooling rate of the first annealing increases the ultimate tensile strength from 1077 MPa to 1229 MPa, but ductility decreases synchronously. With increasing second annealing temperature, the strength first increases and then decreases, while the ductility changes gently. The optimized heat treatment schedule is determined as 880 °C/2 h/AC + 650 °C/4 h/AC.
3. Impact toughness is governed by the combined effects of crack propagation path, interfacial dislocation pile-up, and void nucleation. Sufficient α_p_ phase and relatively thick α_s_ lamellae can induce multiple crack deflections and prolong the propagation path. Rapid cooling leads to consistent crystallographic orientations between α colonies and α_p_ phase, resulting in straight crack propagation and a decrease in impact toughness. Dislocations mainly pile-up at α/β interfaces to induce void formation. The {$10\overset{-}{1}2$} <$11\overset{-}{2}0$> tensile twin acts only as an auxiliary deformation mechanism and contributes limitedly to toughness.
4. After optimized double annealing, the Ti62F alloy exhibits a superior comprehensive balance of strength (1077 MPa), plasticity (16.5%) and toughness (51.5 J) compared with conventional medium-strength titanium alloys such as TA15, TC4, and TC4-DT, showing promising application potential in aerospace structural components. There are limitations in simulating real service conditions, which will be further studied in future work.

## Figures and Tables

**Table 1:** Chemical composition of Ti62F titanium alloy (wt.%).

Al	Mo	V	Cr	Sn	Zr	Ti
5.0~6.5	2.0~4.0	1.0~2.5	1.0~2.5	0.5~2.5	0.5~2.5	Bal.

## Data Availability

The original contributions presented in this study are included in the article. Further inquiries can be directed to the corresponding authors.
